# Clustering patterns of behavioral and metabolic risk factors for noncommunicable diseases in Iran: findings from a national STEPS survey

**DOI:** 10.1080/16549716.2026.2712185

**Published:** 2026-08-10

**Authors:** Mohammad Amin Kharaghani, Safdar Masoumi, Arash Bagherian Ghotbi, Yosra Azizpour, Mina Mirzad, Sepehr Khosravi, Mostafa Moghimi Kheirabady, Moez Shaabanian, Nazila Rezaei, Mahbube Ebrahimpur, Ali Golestani

**Affiliations:** a Non-Communicable Diseases Research Center, Endocrinology and Metabolism Population Sciences Institute, Tehran University of Medical Sciences, Tehran, Iran; b School of Medicine, Tehran University of Medical Sciences, Tehran, Iran; c Department of Biostatistics, Faculty of Medical Sciences, Tarbiat Modares University, Tehran, Iran; d Endocrinology and Metabolism Research Center, Endocrinology and Metabolism Clinical Sciences Institute, Tehran University of Medical Sciences, Tehran, Iran; e Elderly Health Research Center, Endocrinology and Metabolism Population Sciences Institute, Tehran University of Medical Sciences, Tehran, Iran

**Keywords:** Noncommunicable diseases, cluster analysis, risk factors, health surveys, Iran

## Abstract

**Background:**

Noncommunicable diseases (NCDs) are the leading cause of mortality in Iran, driven by behavioral and metabolic risk factors that frequently co-occur.

**Objective:**

To identify patterns of co-occurring behavioral and metabolic NCD risk factors among Iranian adults and characterize their demographic and socioeconomic correlates.

**Methods:**

This cross-sectional study analyzed data from 16,618 adults aged ≥25 years who participated in Iran’s 2021 nationally representative STEPS survey. Thirteen behavioral and metabolic variables, including physical activity, nutrition score, smoking frequency, alcohol intake, salt intake, body mass index, blood pressure, fasting plasma glucose, and lipid markers, were entered into a *K*-means clustering analysis. Clusters were characterized by their risk profiles and demographic/socioeconomic attributes. Multinomial logistic regression examined associations between cluster membership and sociodemographic factors.

**Results:**

Five distinct behavioral-metabolic clusters emerged. The smokers–drinkers (SD) cluster (3.1%) comprised mostly older, less-educated men with high smoking and alcohol use. The healthy-low-risk (HLR) cluster (40.3%) showed favorable profiles and included younger, more educated individuals. The physically active (PA) cluster (6.6%) was characterized mainly by younger men with markedly high physical activity levels. The dyslipidemic (DLP) cluster (26.0%) exhibited high dyslipidemia and overweight prevalence, while the hypertensive-diabetic (HTD) cluster (24.0%) had the highest obesity, hypertension, and diabetes rates, common among older urban adults.

**Conclusion:**

Behavioral and metabolic NCD risk factors in Iran formed five distinct co-occurrence patterns. Nearly half of adults belonged to metabolically high-risk clusters, highlighting the need for targeted prevention strategies that combine lifestyle interventions with screening and management of obesity, hypertension, diabetes, and dyslipidemia.

## Paper Context

- **Main findings:** Five distinct patterns of co-occurring behavioral and metabolic risk factors were identified among Iranian adults, with nearly half of the population concentrated in high-risk groups characterized by obesity, hypertension, diabetes, and abnormal blood lipids.
- **Added knowledge:** This study moves beyond single risk factor analysis by demonstrating how lifestyle behaviors and metabolic abnormalities cluster within specific demographic and socioeconomic groups, revealing population segments that require tailored prevention strategies.
- **Global health impact for policy and action:** Cluster-based risk profiling provides a practical framework for policymakers to design targeted, integrated prevention programs that address overlapping risk factors simultaneously, improving efficiency and reducing the long-term burden of chronic diseases in middle-income countries.

## Background

Noncommunicable diseases (NCDs) such as cardiovascular disease, diabetes, cancer, and chronic respiratory diseases have become the leading causes of mortality worldwide [1]. NCDs accounted for 64.79% of all deaths and 59.88% of disability-adjusted life years (DALYs) in 2021, which is more than all other causes combined [2]. Major NCDs are largely driven by a few well-documented behavioral and metabolic risk factors. Key behavioral risks include tobacco and alcohol use, unhealthy diets (such as low intake of fruits and vegetables and high consumption of salt, fat, and sugar), and physical inactivity. Metabolic risks include conditions like elevated blood pressure (BP), obesity, dyslipidemia, and diabetes. Worldwide, behavioral and metabolic risk factors are attributed to 34% and 41% of NCD-related deaths and account for 25% and 26% of NCD-related DALYs, respectively [3].

Iran is facing NCDs as the major health problem of the country [4]. According to the 2021 national survey of noncommunicable disease risk factor surveillance (STEPS) in Iran, both behavioral and metabolic risk factors for NCDs are highly prevalent. Among behavioral risk factors, 14.0% of adults were current tobacco smokers [5], 6.9% reported lifetime alcohol consumption [6], 51.3% engaged in insufficient physical activity [7], and nearly 98% were salt overconsumers [8]. In terms of metabolic risk factors, 24.96% of the population was obese [9], 14.2% had diabetes [10], 32.0% had hypertension [11], and an alarming 81.0% had dyslipidemia [12]. Moreover, in comparison to the 2016 STEPS study, the prevalence of most of these risk factors has remained constant or shown an increasing trend [13], with the most notable rise observed in diabetes, which has nearly increased 30% over this period [14]. These findings highlight the widespread and multifaceted nature of behavioral and metabolic risks within the Iranian population.

Today’s risk factors are the foundation of tomorrow’s NCDs. These risk factors are easier to identify before they progress into chronic conditions, which require long-term treatment and place a heavy burden on health-care systems. These risk factors are lifestyle-related and modifiable, so early detection, targeted interventions, and behavioral changes are needed and crucial [15]. Studying one risk factor at a time may miss how they interact, influence, and coexist with each other. To get a clear picture of the population risk profile, it is important to consider all risk factors together [16]. Contemporary public health frameworks increasingly emphasize the concepts of risk aggregation, clustering, and multimorbidity, recognizing that these risk factors rarely occur independently; instead, they interact synergistically, compounding long-term damage [17,18]. Thus, there is a need for comprehensive approaches that consider multiple-risk factors simultaneously for a better understanding of the complex nature of NCD risk factors [16].

Although several countries, including Bangladesh and Kenya, have investigated patterns of co-occurring NCD risk factors, evidence from the Middle East remains very limited. A nationally representative study in Bangladesh reported that more than one-third of adults had at least three concurrent NCD risk factors, being more common among older adults, men, and urban residents [15]. Similarly, a study in Kenya identified five distinct clusters of NCD risk factors, including groups characterized by hypertension, obesity, and harmful lifestyle behaviors, and found that age, gender, education, and wealth were important predictors of cluster membership [16]. In the Middle East, only limited evidence exists, including a recent study from Jordan that demonstrated frequent aggregation of behavioral risk factors, such as smoking, physical inactivity, and poor diet across different demographic groups [19]. However, to date, no other studies have examined the clustering of NCD risk factors in the Middle East, particularly in Iran.

*K*-means clustering is an unsupervised statistical method that identifies subgroups within a population based on similarity across selected quantitative variables. This approach allows individuals with similar behavioral and metabolic profiles to be grouped together without predefining risk categories. By applying this approach to NCD risk factors, it becomes possible to capture the patterns of co-occurrence and interaction among multiple risks, revealing how specific factors tend to cluster together in different groups. This understanding facilitates the design of more precise and effective intervention strategies tailored to the unique risk profiles of each subgroup [20].

Unlike previous STEPS analyses, which have primarily focused on the prevalence and trends of individual risk factors or the simple accumulation of multiple risks, this study aimed to identify distinct NCD risk profiles and patterns of co-occurrence among Iranian adults using *K*-means cluster analysis based on the 2021 national STEPS survey data. Recognizing how behavioral and metabolic risks combine across population subgroups provides a more holistic view of the NCD burden and will empower policymakers and public health authorities to develop targeted, evidence-based interventions addressing the most vulnerable groups, ultimately reducing the overall impact of NCDs in Iran.

## Methods

### Study design and participants

**Figure 1.**
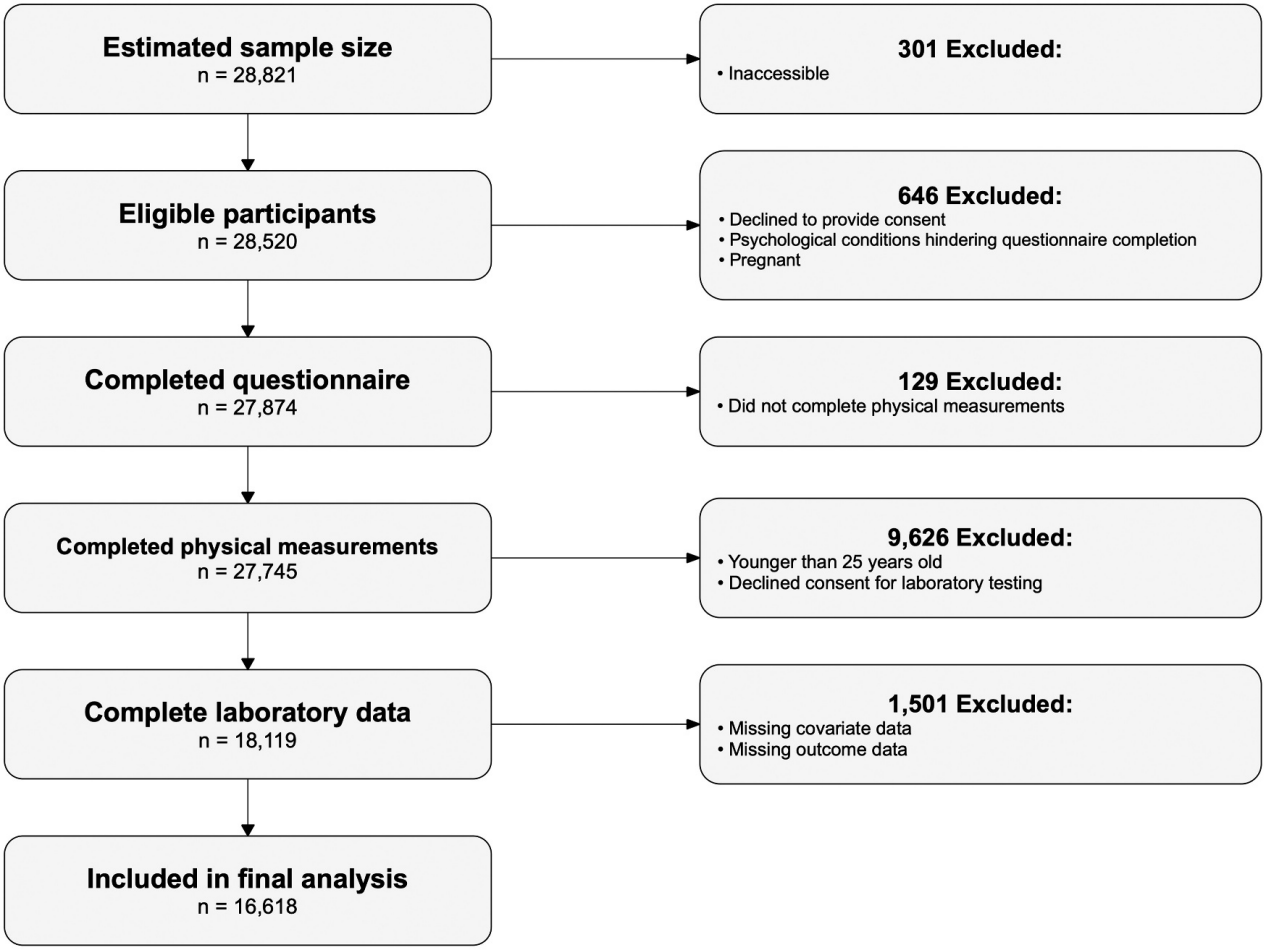
Participant flowchart showing the selection process from the initial STEPS 2021 sample to the final analytical sample.

The study’s data came from the nationally and subnationally representative Iran STEPS 2021 survey [21]. This survey used the WHO STEPwise approach for NCD risk factor surveillance. This study used a three-step process: questionnaires, anthropometric measurements, and laboratory assessments. Systematic cluster sampling was used to obtain a sample of participants aged 18 years and older from rural and urban areas across all 31 provinces of Iran. Of the estimated sample size of 28,821 participants, 301 individuals were inaccessible, leaving 28,520 eligible participants (Figure 1). Among these, 646 were excluded because they declined to provide consent, had psychological conditions that hindered questionnaire completion, or were pregnant. Consequently, 27,874 participants successfully completed the questionnaire.

Among questionnaire respondents, 129 participants did not complete the physical measurements, leaving 27,745 individuals eligible for further assessment. During the laboratory assessment stage, an additional 9626 individuals were excluded because they were younger than 25 years or declined consent for laboratory testing, resulting in 18,119 participants with complete laboratory data. Finally, after excluding 1501 individuals with missing covariate or outcome data, a total of 16,618 participants with complete data across all study variables were included in the final analysis [21]. Comparisons between included and excluded participants are provided in Supplementary Tables S1 and S2.

The first steps of the STEPs survey used a structured questionnaire to gather data on demographics, diet, medical history, physical activity, health quality of life, lifestyle counseling, tobacco and alcohol use, and household assets. In the second step, physical measurements were conducted, including height, weight, and blood pressure. Height was measured with a standard meter while participants stood upright against a wall, ensuring their heels, hips, and the back of their head were properly aligned. Weight was taken using a calibrated Inofit digital scale, with participants dressed in light clothing and without shoes. Before blood pressure measurement, participants rested seated for 15 min. Blood pressure was recorded three times at 3-min intervals using a standard Beurer sphygmomanometer, and the average of the second and third readings was used as the final value. The third phase included laboratory tests such as total serum cholesterol (TC), high-density lipoprotein cholesterol (HDL-C), serum triglycerides (TG), and fasting plasma glucose (FPG), urine sodium, and urine creatinine. All samples were collected in the morning, transported, and stored under cold-chain conditions to a central laboratory for analysis.

### Definition of variables

Several demographic variables were considered, including sex, age (categorized as 25 to 40, 41 to 60, and >60), place of residence (urban/rural), marital status (single (never married), married, divorced/separated with partner, widowed), and having health insurance (no, basic, basic, and complementary). Education level was defined by the number of completed school years, grouped as 0, 1–6, 7–11, and ≥12 years. The wealth index was derived using principal component analysis (PCA) based on ownership of several household asset items [22] and was divided into five quintiles, ranging from the poorest (quintile 1) to the wealthiest (quintile 5), consistent with the Iran STEPS survey methodology [21]. Occupation status was categorized into four groups: employed, unemployed, retired, and unpaid work.

Physical activity was assessed using the Global Physical Activity Questionnaire (GPAQ, version 2) [23], which measured the minutes spent per week in different types of physical activity (vigorous and moderate work, travel, vigorous and moderate recreational activities). These data were converted into Metabolic Equivalent (MET) minutes per week, and low physical activity was considered as <600 MET-min per week [7]. Current smoking and alcohol use were defined as any tobacco use or alcohol consumption within the past 12 months [5]. The nutritional behavior of individuals was assessed by constructing a composite variable. For this purpose, 13 dietary components were considered: number of meals per day, number of snacks per day, days of eating breakfast per week, fruit servings per day, vegetable servings per day, consumption of dairy products (including milk, yogurt, and cheese), type of dairy, red meat, fish, processed meats, whole grains (including brown rice, grain bread, barley bread, and pasta), sugar-sweetened beverages, and nuts and seeds consumption. Each component was scored from 0 to 1 based on participants’ responses, reflecting adherence to dietary guidelines and expert recommendations (Supplementary Table S3). PCA was then applied to these components, and the score of the first principal component was considered the nutrition score. The first principal component explained 33% of the total dietary variance. Individuals were categorized into tertiles according to their nutrition score.

Body mass index (BMI) was calculated by dividing weight in kilograms (kg) by the square of height in meters (m^2^) and classified as ≤25 kg/m^2^ (underweight/normal), 25 to ≤30 kg/m^2^ (overweight), and 30 kg/m^2^ or higher (obese) [9]. Hypertension was defined as systolic blood pressure (SBP) ≥140 mmHg, or diastolic blood pressure (DBP) ≥90 mmHg, or the use of any antihypertensive medication [24].

Diabetes was defined as FPG ≥ 126 mg/dL (7.0 mmol/L) or taking oral antihyperglycemic drugs/insulin based on self-reports. Dyslipidemia was defined as the presence of any abnormal lipid component, including TC ≥ 200 mg/dL and/or TG ≥ 150 mg/dL and/or serum low-density lipoprotein cholesterol (LDL-C) (calculated from the Friedewald formula) ≥130 mg/dL and/or non-HDL-C ≥ 160 mg/dL (subtraction of HDL-C from total cholesterol) and/or HDL-C <40 mg/dL in males and <50 mg/dL in females or taking any lipid-lowering drugs [12]. Salt intake was estimated using spot urine sodium and creatinine levels, applying the Tanaka equation [8]. Salt overconsumption was defined based on the WHO recommendation of salt intake <5 g/day [25].

### Statistical methods

Data cleaning and weighting were performed according to the STEPS 2021 study protocol [21]. To make sure our results represent the national and provincial populations, we used a four-step weighting process: (1) adjusting for people who did not respond overall, (2) adjusting for non-response at each survey step, (3) weighting by age, sex, and area within each province, and (4) final calibration using all previous weights. All reported prevalence rates are weighted and include 95% confidence intervals (CIs).

We used the *K*-means unsupervised clustering algorithm to group participants based on 13 health-related quantitative variables: physical activity (MET-min per week), nutrition score, smoking frequency (mean number of cigarette-equivalent per day), alcohol intake (mean g/day) [6], salt intake, BMI, SBP, DBP, FPG, TC, HDL-C, LDL-C, and TG. These variables have well-established relevance to NCDs and comprehensively capture both behavioral and metabolic risk factors. Additionally, they were available and consistently measured in the STEPS 2021 dataset. To ensure comparability across variables with diverse measurement units and to mitigate the undue influence of differing measurement ranges and potential outliers, all continuous variables were standardized using min–max scaling to a 0–1 range prior to *K*-means clustering. The best number of clusters was chosen using the jump method, a statistical technique that determines the optimal number of clusters by identifying the largest drop (jump) in within-cluster variation as the number of clusters increases [26] and confirmed by visually inspecting the scree plot. For initialization, we employed the ‘k-means++’ method, which automatically selects optimal initial centroids. The analysis was run with 100 iterations, and the convergence criteria were set based on a minimum change in cluster centers (tolerance) to ensure convergence to a stable solution. To evaluate the quality of the *K*-means clustering, we employed the Silhouette Score, a metric that assesses both cohesion within clusters and separation between them. We described each cluster by its main risk factors and summarized participant characteristics within clusters using weighted prevalence and 95% CIs. Differences between groups were considered statistically significant if their 95% CIs did not overlap. To explore the relationship between participant characteristics and cluster membership, we performed multinomial logistic regression. For the univariate analysis, we calculated crude odds ratios (ORs) and 95% CIs for each variable, using healthy and low-risk (HLR) cluster as the reference group. This cluster was selected as the reference out of the five total clusters, because it exhibited the most favorable behavioral and metabolic risk profile, providing a logical clinical baseline for comparison with higher risk clusters. Variables included sex, age, residency, education, wealth index, marital status, occupation, and insurance status. In the multivariable analysis, all variables were included together to estimate adjusted odds ratios (aORs) and 95% CIs, controlling for confounding factors. Multicollinearity among the predictor variables (sex, age, residency, education, wealth index, marital status, occupation, and insurance status) was assessed using VIFs, with all values below 5. The likelihood ratio test confirmed a statistically significant overall model fit compared to the null model. Missing data were handled using complete case analysis to maintain internal validity. All analyses were done using Stata version 17 and R version 4.3.1 or later, with statistical significance set at *p* < 0.05.

## Results

### Characteristics of the study population

Overall, 16,618 participants were included in this study. The proportion of females was higher, accounting for 56.2%. Age distribution showed that 34.0% were 25–40 years, 43.9% were 41–60 years, and 22.1% were above 60. Education levels differed notably: 40.5% of males had 12 or more years of schooling compared to 31.6% of females, while zero education was nearly twice as common among females (22.4%) than males (12.0%). Other demographic details, including wealth, marital status, and employment, are presented in Table 1.

**Table 1.** Baseline demographic and socioeconomic characteristics of the study population.

Variables	Female ( *n* = 9213)	Male ( *n* = 7405)	Total ( *n* = 16,618)
Age			
25–40	3259 (35.4)	2391 (32.3)	5650 (34.0)
41–60	4116 (44.7)	3173 (42.9)	7289 (43.9)
Above 60	1838 (20.0)	1841 (24.9)	3679 (22.1)
Residency			
Rural	2980 (32.4)	2362 (31.9)	5342 (32.2)
Urban	6233 (67.7)	5043 (68.1)	11276 (67.9)
Education			
Zero	2054 (22.4)	883 (12.0)	2937 (17.8)
1–6 years	2751 (30.0)	1869 (25.4)	4620 (28.0)
7–11 years	1456 (15.9)	1624 (22.1)	3080 (18.7)
12 years and over	2897 (31.6)	2983 (40.5)	5880 (35.6)
Wealth index			
Poorest	2133 (23.2)	1420 (19.2)	3553 (21.4)
Class 1	1898 (20.6)	1326 (17.9)	3224 (19.4)
Class 2	1891 (20.5)	1674 (22.6)	3565 (21.5)
Class 3	1767 (19.2)	1608 (21.7)	3375 (20.3)
Class 4	1524 (16.5)	1377 (18.6)	2901 (17.5)
Marital status			
Single	683 (7.4)	598 (8.1)	1281 (7.7)
Married	7185 (78.0)	6623 (89.4)	13,808 (83.1)
Divorced/separate with partner	282 (3.1)	94 (1.3)	376 (2.3)
Widow	1063 (11.5)	90 (1.2)	1153 (6.9)
Occupation			
employed	992 (10.8)	5127 (69.7)	6119 (37.1)
Unpaid work	7767 (84.8)	128 (1.7)	7895 (47.8)
Retired	234 (2.6)	1426 (19.4)	1660 (10.1)
Unemployed	165 (1.8)	678 (9.2)	843 (5.1)
Insurance			
No	640 (7.0)	634 (8.7)	1274 (7.7)
Basic	5960 (65.3)	4658 (63.6)	10,618 (64.5)
Basic + complementary	2530 (27.7)	2035 (27.8)	4565 (27.7)

### Cluster analysis of NCD risk factors

Using 13 risk variables, the optimum number of clusters was determined to be five. The analysis yielded a mean Silhouette Score of 0.43, indicating a moderate level of cluster separation. Smokers–drinkers (SD), comprising 3.1% of participants and totaling 509 individuals; healthy and low risk (HLR), 40.3% with 6700 individuals; physically active (PA), 6.6% or 1101 individuals; dyslipidemic (DLP), 26.0% with 4326 individuals; and hypertensive diabetics (HTD), 24.0% with 3982 individuals (Table 2).

**Table 2.** Mean values of behavioral and metabolic risk factors across the five identified clusters.

	Cluster 1 (SD) *n* = 509	Cluster 2 (HLR) *n* = 6700	Cluster 3 (PA) *n* = 1101	Cluster 4 (DLP) *n* = 4326	Cluster 5 (HTD) *n* = 3982
Physical activity	3312.5 [2746.9, 3878.1]	2584.6 [2460.4, 2708.8]	13,689.2 [12,946.7, 14,431.8]	2476.0 [2331.8, 2620.2]	2317.3 [2174.3, 2460.4]
Nutrition score	0.26 [0.20, 0.33]	0.40 [0.38, 0.42]	0.37 [0.32, 0.41]	0.45 [0.43, 0.48]	0.36 [0.33, 0.38]
smoking frequency	27.84 [24.83, 30.84]	1.52 [1.33, 1.71]	2.22 [1.71, 2.73]	1.49 [1.24, 1.73]	1.42 [1.19, 1.65]
alcohol intake	2.59 [1.26, 3.92]	0.11 [0.08, 0.15]	0.15 [0.05, 0.25]	0.14 [0.09, 0.20]	0.12 [0.07, 0.16]
Salt intake	9.53 [9.30, 9.76]	9.42 [9.35, 9.49]	10.12 [9.96, 10.29]	9.74 [9.66, 9.82]	10.12 [10.03, 10.22]
BMI	26.39 [25.85, 26.92]	26.19 [26.07, 26.32]	26.18 [25.86, 26.50]	27.80 [27.64, 27.96]	29.52 [29.33, 29.72]
FPG	108.10 [102.40, 113.80]	99.69 [98.89, 100.50]	99.57 [97.61, 101.53]	104.47 [103.27, 105.67]	118.49 [116.41, 120.56]
SBP	129.61 [127.51, 131.72]	120.17 [119.69, 120.65]	124.70 [123.54, 125.86]	127.98 [127.30, 128.66]	139.32 [138.46, 140.18]
DBP	78.49 [77.12, 79.86]	74.68 [74.37, 74.99]	78.36 [77.63, 79.09]	78.70 [78.25, 79.15]	85.66 [85.13, 86.18]
TC	171.47 [166.77, 176.17]	158.12 [157.11, 159.12]	165.15 [162.78, 167.52]	200.20 [198.56, 201.83]	165.87 [164.56, 167.17]
HDL-C	40.39 [39.19, 41.59]	43.42 [43.06, 43.77]	41.17 [40.40, 41.94]	43.45 [43.03, 43.87]	38.90 [38.52, 39.28]
LDL-C	99.12 [94.98, 103.26]	89.13 [88.26, 89.99]	95.47 [93.23, 97.70]	123.80 [122.42, 125.19]	90.82 [89.62, 92.02]
TG	159.77 [150.12, 169.42]	127.86 [125.26, 130.46]	142.56 [136.27, 148.85]	164.71 [161.42, 168.00]	180.74 [175.85, 185.63]
Cluster size (%)	3.1	40.3	6.6	26	24

SD, smokers–drinkers; HLR, healthy and low risk; PA, physically active; DLP, dyslipidemic; HTD, hypertensive diabetics; BMI, body mass index; FPG, fasting plasma glucose; SBP, systolic blood pressure; DBP, diastolic blood pressure; TC, total cholesterol; HDL-C, high-density lipoprotein cholesterol; LDL-C, low-density lipoprotein cholesterol; TG, triglycerides.

Smokers–drinkers (SD) included the highest prevalence of currently smoking (61.1%, 95% CI: 56.2–65.7) and currently alcoholic (10.1%, 95% CI: 7.4–13.6). Individuals in this group reported a mean smoking frequency of 27.8 cigarette-equivalent per day (95% CI: 24.8–30.8), statistically significantly higher than in other clusters, where smoking frequencies ranged from 1.4 to 2.2 cigarette-equivalent/day. Similarly, the mean alcohol intake in the SD cluster was 2.6 g/day (95% CI: 1.3–3.9), compared to a range of 0.11 to 0.15 g/day in the other clusters. Other notable health risks in this group included diabetes (16.7%, 95% CI: 12.6–21.8), ranking second highest among all clusters; hypertension (41.8%, 95% CI: 35.9–47.8), also the second highest; and dyslipidemia (89.3%, 95% CI: 85.7–92.1), which ranked third highest (Tables 2 and 3).

**Table 3.** Prevalence of key behavioral and metabolic risk factor conditions across the five identified clusters.

	Cluster 1 (SD) *n* = 509	Cluster 2 (HLR) *n* = 6700	Cluster 3 (PA) *n* = 1101	Cluster 4 (DLP) *n* = 4326	Cluster 5 (HTD) *n* = 3982
Low physical activity	46.6 [41.7, 51.5]	47.7[46.3,49.0]	16.5[14.2,19.1]	46.4[44.8,48.1]	49.0[47.2,50.7]
Currently smoking	61.1[56.2,65.7]	13.5[12.6,14.4]	17.3[14.9,20.0]	11.6[10.5,12.7]	11.9[10.8,13.1]
Currently alcoholic	10.1[7.4,13.6]	3.2[2.7,3.7]	4.3[3.1,6.0]	2.8[2.3,3.5]	3.3[2.7,4.0]
Nutrition tertile 1	15.0[12.5,17.9]	13.7[13.0,14.5]	14.5[12.8,16.4]	12.5[11.7,13.5]	15.2[14.2,16.2]
Nutrition tertile 2	39.6[34.1,45.4]	32.4[30.9,34.0]	34.7[31.2,38.4]	30.3[28.5,32.2]	31.5[29.5,33.6]
Nutrition tertile 3	45.4[39.4,51.5]	53.8[52.2,55.5]	50.8[46.9,54.7]	57.1[55.1,59.2]	53.3[51.1,55.5]
Salt overconsumption	98.0[96.1,99.0]	97.5[97.1,97.9]	98.1[97.0,98.9]	98.4[97.9,98.7]	98.3[97.8,98.7]
Normal weight	40.3[35.6,45.2]	38.5[37.2,39.8]	39.7[36.5,42.9]	27.3[25.8,28.8]	18.2[16.9,19.6]
Overweight	34.5[30.0,39.4]	38.5[37.2,39.8]	36.8[33.7,40.1]	42.3[40.6,43.9]	38.6[36.9,40.3]
Obesity	22.0[18.2,26.4]	19.1[18.1,20.2]	20.1[17.5,22.9]	28.9[27.4,30.4]	42.4[40.7,44.2]
Diabetes	16.7[12.6,21.8]	9.7[8.7,10.8]	8.1[6.1,10.8]	11.3[10.2,12.6]	26.7[24.8,28.8]
Hypertension	41.8[35.9,47.8]	21.3[20.0,22.8]	28.3[24.7,32.1]	36.4[34.4,38.5]	62.8[60.7,64.9]
Dyslipidemia	89.3[85.7,92.1]	78.3[76.8,79.8]	82.9[80.1,85.5]	95.2[94.4,95.9]	93.0[91.9,93.9]

SD, smokers–drinkers; HLR, healthy and low risk; PA, physically active; DLP, dyslipidemic; HTD, hypertensive diabetics.

Healthy and low risk (HLR) was the largest cluster, representing 40.3% of the total population. The prevalence of currently smoking and currently alcoholic was relatively low (13.5%, 95% CI: 12.6–14.4; and 3.2%, 95% CI: 2.7–3.7, respectively). Additionally, this cluster had the lowest prevalence among all five clusters for obesity (19.1%, 95% CI: 18.1–20.2), hypertension (21.3%, 95% CI: 20.0–22.8), and dyslipidemia (78.3%, 95% CI: 76.8–79.8). Its prevalence of diabetes was also comparatively low, ranked fourth among the clusters (9.7%, 95% CI: 8.7–10.8), closely following the PA cluster. Based on Table 2, this cluster ranked either first or second in a healthier direction across nearly all behavioral and metabolic indicators. It had the lowest systolic (120.17 mmHg, 95% CI: 119.69–120.65) and diastolic (74.68 mmHg, 95% CI: 74.37–74.99) blood pressure, total cholesterol (158.12 mg/dL, 95% CI: 157.11–159.12), and triglycerides (127.86 mg/dL, 95% CI: 125.26–130.46), each ranking first. It also ranked second in BMI (26.19, 95% CI: 26.07–26.32), FPG (99.69 mg/dL, 95% CI: 98.89–100.50), LDL-C (89.13 mg/dL, 95% CI: 88.26–89.99), HDL-C (43.42 mg/dL, 95% CI: 43.06–43.77), smoking frequency (1.52 cigarette-equivalent/day, 95% CI: 1.33–1.71), alcohol intake (0.11 g/day, 95% CI: 0.08–0.15), salt intake (9.42 g/day, 95% CI: 9.35–9.49), and nutrition score (0.40, 95% CI: 0.38–0.42). The only exception was total physical activity, where it ranked third (2584.6 MET-min/week, 95% CI: 2460.4–2,708.8). This supports the classification of this cluster as the healthiest subgroup (Tables 2 and 3).

Physically active (PA) exhibited the lowest prevalence of physical inactivity at 16.5% (95% CI: 14.2–19.1). It was in strong contrast to the other clusters, where the prevalence ranged from 46.4% to 49.0%. Correspondingly, the PA cluster recorded the highest average physical activity level at 13,689.2 MET-min/week (95% CI: 12,946.7–14,431.8). This level of activity far exceeded the levels observed in the other clusters, which ranged from 2317.3 to 3312.5 MET-min/week. This exceptionally high activity was driven by leadership across all measured physical activity subtypes. The most dramatic difference was seen in vigorous work, where the PA cluster’s mean of 7953.8 MET-min/week was nearly eight times higher than any other group and represented an absolute difference of over 6900 MET-min/week compared to the next highest cluster. This group also led statistically significantly in moderate work (3337.9 MET-min/week), transport activity (1737.9 MET-min/week), and both vigorous (1019.5 MET-min/week) and moderate (338.5 MET-min/week) recreational activities (Supplementary Tables S4 and S5). This cluster also had the lowest prevalence of diabetes among all clusters (8.1%, 95% CI: 6.1–10.8). Additionally, following the HLR cluster, it had the second lowest prevalence of obesity (20.1%, 95% CI: 17.5–22.9), hypertension (28.3%, 95% CI: 24.7–32.1), and dyslipidemia (82.9%, 95% CI: 80.1–85.5) (Tables 2 and 3).

Dyslipidemic (DLP) had the highest prevalence of dyslipidemia among all clusters (95.2%, 95% CI: 94.4–95.9), as well as the highest prevalence of overweight (42.3%, 95% CI: 40.6–43.9). It also ranked second in obesity prevalence (28.9%, 95% CI: 27.4–30.4). Consistent with its clinical profile, the DLP cluster exhibited the most unfavorable lipid values. TC and LDL-C were highest in this cluster, averaging 200.2 mg/dL (95% CI: 198.6–201.8) and 123.8 mg/dL (95% CI: 122.4–125.2), respectively. Despite having a relatively favorable HDL-C level of 43.5 mg/dL (95% CI: 43.0–43.9), the DLP cluster reported the second highest triglyceride (TG) concentration, averaging 164.7 mg/dL (95% CI: 161.4–168.0). In contrast, this cluster showed the lowest prevalence of currently smoking (11.6%, 95% CI: 10.5–12.7) and currently alcoholic (2.8%, 95% CI: 2.3–3.5) (Tables 2 and 3).

Hypertensive diabetics (HTD) represented the highest prevalence among all clusters for obesity (42.4%, 95% CI: 40.7–44.2), diabetes (26.7%, 95% CI: 24.8–28.8), and hypertension (62.8%, 95% CI: 60.7–64.9). Additionally, this cluster ranked second in dyslipidemia prevalence at 93.0% (95% CI: 91.9–93.9). This cluster had the highest mean values for BMI (29.52, 95% CI: 29.33–29.72), FPG (118.49 mg/dL, 95% CI: 116.41–120.56), SBP (139.32 mmHg, 95% CI: 138.46–140.18), and DBP (85.66 mmHg, 95% CI: 85.13–86.18) across all clusters (Tables 2 and 3).

### Profile of the NCD risk clusters

The five clusters exhibited distinct demographic profiles (Table 4). HLR and DLP clusters had the highest proportion of females, with 56.8% (95% CI: 55.5–58.1) and 58.2% (95% CI: 56.6–59.9), respectively. In contrast, the PA cluster had the highest proportion of males at 58.7% (95% CI: 55.4–61.9).

**Table 4.** Demographic and socioeconomic profile of participants within each NCD risk cluster.

Variables	Cluster 1 (SD)	Cluster 2 (HLR)	Cluster 3 (PA)	Cluster 4 (DLP)	Cluster 5 (HTD)
Sex					
Female	44.7 [39.9, 49.6]	56.8[55.5,58.1]	41.3[38.1,44.6]	58.2[56.6,59.9]	55.6[53.9,57.4]
Male	55.3[50.4,60.1]	43.2[41.9,44.5]	58.7[55.4,61.9]	41.8[40.1,43.4]	44.4[42.6,46.1]
Age					
25–40	16.0[12.7,19.8]	44.9[43.5,46.2]	43.6[40.4,46.9]	25.1[23.7,26.5]	21.7[20.3,23.1]
41–60	52.7[47.8,57.6]	37.5[36.2,38.8]	43.7[40.4,47.0]	50.2[48.6,51.9]	48.9[47.2,50.7]
Above 60	31.3[26.9,36.1]	17.7[16.7,18.7]	12.7[10.7,15.1]	24.7[23.3,26.1]	29.4[27.8,31.0]
Residency					
Rural	36.1[31.5,40.9]	28.9[27.8,30.1]	46.9[43.6,50.2]	30.1[28.6,31.6]	27.4[25.9,28.9]
Urban	63.9[59.1,68.5]	71.1[69.9,72.2]	53.1[49.8,56.4]	69.9[68.4,71.4]	72.6[71.1,74.1]
Education					
Zero	26.8[22.7,31.3]	12.7[11.9,13.6]	14.6[12.5,17.0]	18.1[16.9,19.4]	21.6[20.2,23.0]
1–6 years	38.8[34.1,43.7]	23.9[22.8,25.1]	31.0[28.0,34.1]	28.8[27.3,30.3]	30.1[28.5,31.8]
7–11 years	16.7[13.4,20.7]	20.0[18.9,21.1]	26.2[23.3,29.3]	17.7[16.4,19.0]	18.1[16.8,19.5]
12 years and over	17.7[14.3,21.8]	43.4[42.0,44.7]	28.3[25.4,31.4]	35.5[33.9,37.1]	30.2[28.6,31.8]
Wealth index					
Poorest	30.9[26.6,35.6]	19.3[18.3,20.4]	25.2[22.4,28.1]	19.5[18.2,20.8]	21.1[19.7,22.5]
Class 1	19.7[16.1,24.0]	20.4[19.4,21.5]	21.0[18.4,23.8]	19.4[18.1,20.8]	19.4[18.0,20.8]
Class 2	22.3[18.4,26.6]	19.7[18.7,20.8]	22.9[20.3,25.8]	19.8[18.6,21.2]	21.5[20.1,23.0]
Class 3	17.6[14.2,21.6]	20.6[19.5,21.7]	18.6[16.1,21.3]	21.3[20.0,22.7]	20.9[19.5,22.4]
Class 4	9.6[7.0,12.9]	19.9[18.9,21.0]	12.4[10.3,14.8]	20.0[18.6,21.3]	17.2[15.9,18.5]
Marital status					
Single	5.8[3.9,8.5]	8.8[8.1,9.6]	4.9[3.7,6.6]	7.5[6.7,8.4]	6.2[5.4,7.1]
Married	85.1[81.3,88.3]	82.5[81.5,83.5]	91.5[89.5,93.1]	80.7[79.3,82.0]	83.5[82.2,84.7]
Divorced/separate with partner	2.3[1.3,4.3]	2.8[2.3,3.2]	1.5[0.9,2.5]	2.6[2.1,3.2]	2.2[1.7,2.8]
Widow	6.7[4.6,9.7]	5.9[5.3,6.6]	2.1[1.4,3.2]	9.2[8.3,10.2]	8.1[7.2,9.1]
Occupation					
Employed	39.3[34.6,44.3]	40.1[38.8,41.5]	54.9[51.6,58.1]	33.4[31.9,35.0]	32.6[31.0,34.2]
Unpaid work	41.9[37.2,46.8]	46.6[45.2,47.9]	37.7[34.5,40.9]	50.5[48.8,52.2]	49.0[47.3,50.8]
Retired	12.5[9.5,16.2]	8.4[7.7,9.2]	4.3[3.2,5.9]	11.2[10.1,12.3]	13.5[12.3,14.7]
Unemployed	6.3[4.3,9.0]	4.9[4.4,5.5]	3.1[2.2,4.4]	4.9[4.2,5.7]	4.9[4.2,5.7]
Insurance					
No	10.4[7.7,14.0]	9.6[8.9,10.5]	10.0[8.2,12.3]	8.3[7.5,9.3]	8.2[7.3,9.2]
Basic	66.2[61.4,70.8]	63.5[62.2,64.7]	76.0[73.0,78.7]	61.4[59.8,63.0]	59.2[57.5,60.9]
Basic + complementary	23.3[19.4,27.8]	26.9[25.7,28.1]	14.0[11.8,16.5]	30.2[28.7,31.8]	32.6[31.0,34.3]

SD, Smokers–drinkers; HLR, healthy and low risk; PA, physically active; DLP, dyslipidemic; HTD, hypertensive diabetics.

Younger individuals aged 25–40 years were most prevalent in the HLR and PA clusters, accounting for 44.9% (95% CI: 43.5–46.2) and 43.6% (95% CI: 40.4–46.9), respectively. In contrast, the prevalence of this age group ranged from 16.0% to 25.1% in other clusters. Meanwhile, older adults (above 60 years) were most common in the SD and HTD clusters, comprising 31.3% (95% CI: 26.9–36.1) and 29.4% (95% CI: 27.8–31.0), respectively. Conversely, their prevalence was lowest in the PA cluster (12.7%, 95% CI: 10.7–15.1) and HLR cluster (17.7%, 95% CI: 16.7–18.7).

Urban residency was higher in the HLR, DLP, and HTD clusters compared to others, with urban proportions of 71.1% (95% CI: 69.9–72.2), 69.9% (95% CI: 68.4–71.4), and 72.6% (95% CI: 71.1–74.1), respectively. The PA cluster had the lowest urban representation at 53.1% (95% CI: 49.8–56.4).

Education and wealth varied statistically significantly across clusters. The HLR cluster had the highest prevalence of individuals with 12 or more years of education (43.4%, 95% CI: 42.0–44.7) and the lowest prevalence in the poorest wealth category (19.3%, 95% CI: 18.3–20.4). In contrast, the SD cluster had the lowest prevalence with 12 or more years of education (17.7%, 95% CI: 14.3–21.8) and the highest prevalence with no formal education (26.8%, 95% CI: 22.7–31.3). It also has the highest prevalence in the poorest wealth category (30.9%, 95% CI: 26.6–35.6).

Employment was most common in the PA cluster, where 54.9% (95% CI: 51.6–58.1) of individuals were employed. In contrast, the highest prevalence of unemployment was observed in the SD cluster, at 6.3% (95% CI: 4.3–9.0).

### Factors associated with NCD risk clusters

The results of univariate analysis are provided in Supplementary Table S6. Multivariable analysis was performed, and Cluster HLR was used as the reference group for all comparisons (Table 5 and Supplementary Table S7). Compared with females, males had statistically significantly higher odds of belonging to the SD (adjusted odds ratio (aOR) = 2.42, 95% CI: 1.50–3.90), PA (aOR = 2.04, 95% CI: 1.49–2.78), and HTD (aOR = 1.24, 95% CI: 1.05–1.47) clusters.

**Table 5.** Multivariable multinomial logistic regression analysis of predictors for NCD risk cluster membership.

Variables	Cluster 1 (SD)	Cluster 2 (HLR)	Cluster 3 (PA)	Cluster 4 (DLP)	Cluster 5 (HTD)
Sex (female)					
Male	**2.42 (1.50, 3.90)**	1 (Reference)	**2.04 (1.49, 2.78)**	1.16 (0.99, 1.36)	**1.24 (1.05, 1.47)**
Age (25–40)					
41–60	**3.40 (2.53, 4.56)**	1 (Reference)	1.11 (0.93, 1.32)	**2.38 (2.13, 2.67)**	**2.43 (2.15,2.73)**
Above 60	**3.33 (2.26,4.92)**	1 (Reference)	0.75 (0.56,1.00)	**2.33 (1.98,2.74)**	**2.63 (2.23,3.11)**
Residency (rural					
Urban	1.03 (0.82,1.30)	1 (Reference)	**0.62 (0.52,0.72)**	0.93 (0.84,1.03)	**1.17 (1.05,1.31)**
Education (zero)					
1–6 years	0.85 (0.64,1.13)	1 (Reference)	0.90 (0.71,1.15)	0.98 (0.85,1.14)	**0.82 (0.71,0.95)**
7–11 years	**0.53 (0.37,0.77)**	1 (Reference)	0.87 (0.66,1.16)	0.88 (0.74,1.04)	**0.72 (0.60,0.86)**
12 years and over	**0.32 (0.22,0.49)**	1 (Reference)	**0.57 (0.42,0.76)**	0.87 (0.73,1.03)	**0.58 (0.49,0.69)**
Wealth index (poorest)					
Class 1	**0.71 (0.53,0.96)**	1 (Reference)	0.92 (0.74,1.15)	1.00 (0.87,1.15)	0.92 (0.79,1.06)
Class 2	0.83 (0.62,1.11)	1 (Reference)	0.91 (0.74,1.14)	1.12 (0.98,1.29)	1.11 (0.96,1.27)
Class 3	0.75 (0.54,1.04)	1 (Reference)	0.85 (0.67,1.08)	**1.20 (1.04,1.39)**	1.07 (0.92,1.25)
Class 4	**0.51 (0.33,0.78)**	1 (Reference)	**0.73 (0.56,0.97)**	**1.19 (1.01,1.39)**	0.93 (0.79,1.10)
Marital status (single)					
Married	**0.58 (0.37,0.90)**	1 (Reference)	**1.71 (1.23,2.39)**	**0.68 (0.57,0.81)**	**0.75 (0.62,0.90)**
Divorced/Separate with partner	0.60 (0.28,1.27)	1 (Reference)	1.00 (0.52,1.92)	0.73 (0.53,1.01)	**0.68 (0.48,0.96)**
Widow	**0.41 (0.22,0.75)**	1 (Reference)	0.72 (0.41,1.26)	0.82 (0.64,1.05)	**0.65 (0.50,0.85)**
Occupation (employed)					
Unpaid work	1.42 (0.87,2.34)	1 (Reference)	1.02 (0.74,1.40)	**1.33 (1.13,1.57)**	**1.28 (1.08,1.53)**
Retired	1.13 (0.77,1.67)	1 (Reference)	**0.61 (0.42,0.88)**	1.14 (0.96,1.36)	**1.24 (1.04,1.48)**
Unemployed	0.78 (0.49,1.22)	1 (Reference)	**0.46 (0.31,0.68)**	1.08 (0.87,1.34)	0.97 (0.78,1.21)
Insurance (no)					
Basic	0.83 (0.57,1.20)	1 (Reference)	0.97 (0.75,1.26)	1.01 (0.85,1.19)	0.99 (0.84,1.18)
Basic + complementary	0.72 (0.46,1.12)	1 (Reference)	**0.62 (0.45,0.85)**	1.00 (0.83,1.20)	1.10 (0.91,1.33)

SD, Smokers–drinkers; HLR, healthy and low risk; PA, physically active; DLP, dyslipidemic; HTD, hypertensive diabetics.

Individuals aged over 40 had statistically significantly higher odds of being classified in the SD, DLP, and HTD clusters. In particular, those aged above 60 had increased odds of belonging to the SD (aOR = 3.33, 95% CI: 2.26–4.92), DLP (aOR = 2.33, 95% CI: 1.98–2.74), and HTD (aOR = 2.63, 95% CI: 2.23–3.11) clusters, compared to younger adults aged 25–40 years.

Urban residents had higher odds of being in the HTD cluster (aOR = 1.17, 95% CI: 1.05–1.31), and lower odds of being in the PA cluster (aOR = 0.62, 95% CI: 0.52–0.72). Higher education (≥12 years) statistically significantly decreased the odds of belonging to the SD (aOR = 0.32, 95% CI: 0.22–0.49), PA (aOR = 0.57, 95% CI: 0.42–0.76), and HTD (aOR = 0.58, 95% CI: 0.49–0.69) clusters compared to having no formal education. Additionally, individuals in the wealthiest group had reduced odds of membership in the SD (aOR = 0.51, 95% CI: 0.33–0.78) and PA (aOR = 0.73, 95% CI: 0.56–0.97) clusters compared to those in the poorest category.

Compared to single individuals, married individuals had lower odds of belonging to SD (aOR = 0.58, 95% CI 0.37–0.90), DLP (aOR = 0.68, 95% CI: 0.57–0.81), and HTD (aOR = 0.75, 95% CI: 0.62–0.90), but higher odds of PA (aOR = 1.71, 95% CI: 1.23–2.39). Widowed individuals had lower odds of SD (aOR = 0.41, 95% CI: 0.22–0.75) and HTD (aOR = 0.65, 95% CI: 0.50–0.85). Compared to employed individuals, those with unpaid work had higher odds of belonging to DLP (aOR = 1.33, 95% CI: 1.13–1.57) and HTD (aOR = 1.28, 95% CI: 1.08–1.53). Retired individuals had lower odds of PA (aOR = 0.61, 95% CI: 0.42–0.88) but higher odds of HTD (aOR = 1.24, 95% CI: 1.04–1.48). Unemployed individuals had lower odds of PA (aOR = 0.46, 95% CI: 0.31–0.68).

## Discussion

The cluster analysis of NCD risk factors within the Iranian population using 2021 national STEPS survey described distinct co-occurrence patterns of behavioral, demographic, and metabolic risk factors, which may inform targeted public health strategies. We identified five distinct clusters, each characterized by the co-occurrence of certain risk factors. Additionally, notable demographic and socioeconomic disparities were observed between these clusters.

The identification of five distinct risk clusters supports previous research that suggests NCD risk factors tend to cluster together rather than occurring independently [27–29]. Clustering is particularly concerning because individuals with multiple risk factors face a higher cumulative risk of developing chronic diseases, such as cardiovascular disease (CVD), diabetes, and hypertension [30].

International evidence supports the tendency of NCD risk factors to cluster, although previous studies differ substantially in data sources, age groups, and analytical approaches. Among the most comparable studies, a Kenyan analysis of the 2015 national STEPS survey used *K*-medians clustering on binary NCD risk variables and identified five risk profiles, including hypertensive, harmful-user, obese, and diet-related clusters; age, sex, education, and wealth were important predictors of cluster membership [16]. These findings are broadly consistent with our identification of distinct metabolic and behavioral clusters, particularly the HTD, DLP, and SD clusters.

Other STEPS-based studies further support the clustering of NCD risks, although they generally used risk-factor counts rather than unsupervised clustering. In Bangladesh, 38% of adults had at least three NCD risk factors, with clustering associated with older age, male sex, urban residence, education, and housing quality [15]. Similarly, a multisite STEPS-based study across rural Asian populations reported that a large proportion of adults had three or more risk factors, with clustering associated with age, male sex, and education [31]. A national STEPS survey in Nepal also showed accumulation of multiple risk factors within individuals, with only 0.4% of the population free from the eight assessed risk factors and a mean of approximately two risk factors per participant [32]. These findings align with our observation that NCD risk is not randomly distributed but concentrates within identifiable demographic and socioeconomic groups. In contrast, non-STEPS studies from Brazil and South Africa, including adolescent cohorts, similarly demonstrated co-occurrence of behavioral or metabolic risks across population subgroups [33,34].

Smokers–drinkers (SD) were predominantly older males, had lower education levels, and included individuals more likely to engage in unhealthy behaviors, such as smoking and alcohol consumption, and exhibited notable metabolic risks, including hypertension, diabetes, and dyslipidemia. These findings are consistent with global evidence showing that tobacco and alcohol use frequently co-occur [35–37]. This dual exposure is strongly linked to increased risks of noncommunicable diseases (NCDs) [38].

Healthy and low risk (HLR) was the largest cluster, characterized by relatively young individuals with higher education and socioeconomic status. This group exhibited a low prevalence of both behavioral and metabolic risk factors; smoking and alcohol use were relatively uncommon, and the prevalence of obesity, hypertension, dyslipidemia, and diabetes were the lowest among all clusters. This profile reflects healthier lifestyle behaviors and more favorable socioeconomic conditions.

The physically active (PA) cluster primarily consisted of younger male individuals, many of whom may work in physically demanding jobs, particularly in rural areas. Their high levels of vigorous and moderate physical activity, which may be related to occupational labor, were accompanied by lower prevalence of metabolic conditions, such as obesity, hypertension, and diabetes. However, this physical resilience does not necessarily equate to overall better health. Their lifestyle, potentially shaped by the demands of rural labor, may also involve contextual challenges, such as limited access to health care [39,40], and inadequate nutrition [41], although these factors were not directly measured. Moreover, probably due to their active engagement in both work and social settings, individuals in this cluster show higher prevalence of behavioral risk factors, such as smoking and alcohol use. These combined factors suggest that the PA cluster represents a distinct population whose health risks and strengths are shaped by complex social and environmental influences. Tailored interventions should not only leverage their physical activity but also address underlying vulnerabilities to prevent a shift toward higher-risk clusters like smokers drinkers.

Dyslipidemic (DLP) cluster consisted of adults and older individuals, has a high prevalence of dyslipidemia and overweight status, with a moderate level of hypertension. Smoking and alcohol use are relatively low in this group. The cluster closely resembles the pattern of metabolic syndrome and requires focused management of lipid abnormalities and weight control to reduce cardiovascular risk and prevent possible progression to the hypertensive fiabetics cluster [42].

Hypertensive diabetics (HTD) cluster had the highest prevalence of obesity, diabetes, and hypertension, primarily affecting older individuals and those from urban areas.

Individuals in the HLR cluster had higher levels of education and wealth, reflecting global trends where higher educational attainment is linked to healthier behaviors and better health outcomes [36,43]. In contrast, SD and HTD, who had a high frequency of risk factors, were more likely to have lower education levels, supporting evidence that limited health literacy and economic barriers contribute to poor lifestyle choices and delayed healthcare access [44].

The clustering of dyslipidemia, hypertension, and obesity in DLP and HTD aligns with the concept of metabolic syndrome, a well-recognized condition that significantly increases the risk of cardiovascular disease and stroke [45,46]. Studies indicate that a combination of high blood pressure, obesity, and high cholesterol levels is more predictive of cardiovascular events than any single risk factor alone, reinforcing the importance of comprehensive risk assessment approaches. In middle-income countries, urbanization and sedentary lifestyles contribute significantly to the increased prevalence of these metabolic risk factors and development of metabolic syndrome [47].

One study from the United States [37] identified a cluster with a high prevalence of male smokers and drinkers, which aligns with our findings of male predominance in the SD cluster. However, their obesity-driven clusters were predominantly female, whereas in our study, the obesity-related clusters showed statistically significant male predominance in the HTD cluster and a statistically nonsignificant male trend in the DLP cluster. This difference may be attributed to lifestyle and cultural factors unique to the Iranian male population, such as lower physical activity or different dietary patterns compared to males in the United States, contributing to the higher metabolic risk.

Based on our clustering results, being married is associated with a lower likelihood of belonging to the SD, DLP, and HTD clusters. This protective association may be largely attributed to the social audit performed by other household members, who monitor and influence one another’s lifestyle choices and eating habits. Consequently, married individuals are less likely to engage in smoking and alcohol consumption and consume unhealthy, low-nutrient foods that trigger diabetes, hypertension, and obesity. Additionally, personal social networks have been shown to promote adherence to healthier behaviors, further supporting the lower prevalence of high-risk behaviors and metabolic conditions among married individuals in our study [48]. However, the opposite explanation is also possible – healthier individuals may be more likely to enter or remain in marriage – an alternative that cannot be distinguished in a cross-sectional study.

In our study, urban residency was statistically significantly and negatively associated with membership in the PA cluster, indicating that individuals in this group are more likely to reside in rural areas. This aligns with the nature of the PA cluster, which largely consists of individuals engaged in physically demanding labour common in rural contexts, such as farming, construction, or manual trades. In contrast to our findings, some studies suggested the potential benefits of urban living for promoting physical activity through infrastructure like parks and organized sports. However, evidence from countries, such as India and Sri Lanka, showed that increasing urbanicity is often associated with lower levels of physical activity and higher prevalence of obesity, diabetes, and other noncommunicable disease (NCD) risk factors [49,50]. In Iran, similar urban challenges, such as sedentary job structures, limited pedestrian infrastructure, environmental stressors like air pollution and traffic congestion, and cultural norms that discourage women’s outdoor activity [51] may be associated with lower physical activity despite the presence of urban amenities. While improving access to physical activity in urban settings is crucial, it is equally important not to overlook the health needs of physically active rural populations. Despite their high levels of occupational activity, these individuals may face other health risks, including poor nutrition [41], limited healthcare access [39,40], and occupational hazards [52]. Thus, differentiated health strategies are needed, ones that reduce structural barriers to activity in urban areas while also supporting the broader well-being of rural laborers who may appear Physically Active but remain vulnerable in other dimensions.

One study using 2016 STEPS data reported the percent changes from 2016 to 2030 for six major risk factors: smoking (−25.55%), physical inactivity (31.45%), salt intake (−13.1%), hypertension (HTN) (37.75%), obesity and overweight (36.2%), and diabetes (17.75%). The findings indicate that four out of these six risk factors are increasing, with hypertension showing the largest increase at 37.75%, closely followed by obesity and overweight at 36.2%. These similar upward trends suggest a strong relationship between hypertension and obesity, which is also reflected in our clustering results. Specifically, our HTD cluster exhibits the highest frequencies of both hypertension and obesity, reinforcing the idea that these two conditions are closely linked and may change concurrently. This is consistent with national STEPS data from 2007 to 2021, which showed statistically significant increases in obesity, diabetes, and metabolic syndrome among Iranian adults with hypertension, particularly in specific subgroups such as males and rural residents [53]. The rising trend in key risk factors highlights the need for early, targeted interventions in clusters like HTD and DLP. Without action, these clusters may continue fueling the rise in noncommunicable diseases. Cluster-based prevention and management strategies, especially where risk factors overlap, can help slow these projections and reduce pressure on the healthcare system over time [13]. Moreover, it is well established that reducing obesity can also lead to significant reductions in hypertension, providing a dual benefit in managing these interconnected risk factors [54,55].

From a public health perspective, the high-risk DLP and HTD clusters, together comprising approximately 50% of the total population, require targeted metabolic risk management interventions. These should include lifestyle modifications, improved access to preventive health care and policy-driven efforts to promote healthier food environments and increased physical activity.

Conversely, the SD cluster, characterized by high smoking and alcohol consumption and predominantly adults aged 40 and above, demonstrates a critical need for earlier behavioral education and intervention targeting younger populations to prevent progression to this cluster. Studies have shown that interventions delivered through digital platforms, such as smartphone apps, text messaging, and online programs, can effectively support behavior change by providing personalized guidance, reminders, and motivation to reduce tobacco and alcohol use [56]. These technology-based approaches increase accessibility and engagement, especially among younger individuals.

Mood and psychological well-being were important across the SD, DLP, and HTD clusters. Previous studies have reported negative emotional states may have contributed to continued substance use in the SD cluster [57]. In addition, mood-related disorders, such as job stress [58], social isolation [59], depression, and anxiety [60], are known to have a bidirectional relationship with metabolic dysfunction, which may explain the higher prevalence of metabolic abnormalities in the DLP and HTD clusters.

Incorporating strategies to address mental health, such as stress management, psychological support may enhance the effectiveness of behavioral interventions in reducing risk factors, not only in the SD cluster [61] but also in preventing and managing metabolic syndrome in the DLP and HTD clusters [62].

This study benefits from using a large, nationally representative dataset collected through the standardized WHO STEPS methodology, enhancing the generalizability of findings to the adult Iranian population. The use of cluster analysis allowed for a comprehensive understanding of how multiple behavioral and metabolic risk factors co-occur and interact within distinct subgroups, providing nuanced insights for targeted public health interventions. However, several methodological limitations should be acknowledged. The cross-sectional design limits causal inference; therefore, the findings should be interpreted as associative rather than causal. This design also prevented the assessment of changes in cluster membership over time. In addition, the K-means algorithm assumes spherical and equally sized clusters, as well as equal variable contributions to distance calculations. Although all variables were standardized before analysis, these assumptions, together with the asymmetric distributions of several behavioral variables, may have influenced cluster stability and interpretability. K-means is also sensitive to initial centroid selection, outliers and data scaling, which can affect the robustness of the results. Additionally, reliance on self-reported behavioral data, such as smoking, alcohol use, and physical activity measured by the Global Physical Activity Questionnaire (GPAQ), may be subject to reporting biases, recall biases, and measurement error, especially in Iranian cultural contexts where substance use is stigmatized. Furthermore, the use of complete-case analysis may have introduced selection bias if excluded participants differed systematically from those included in the final analytic sample. Another limitation of this approach is that some metabolic variables are biologically related and may be correlated. Because *K*-means clustering relies on straight-line distances, the simultaneous inclusion of multiple related metabolic markers may increase the relative influence of the metabolic domain on cluster formation. Finally, some potentially relevant risk factors, including detailed dietary information and mental health indicators, such as stress, depression, and anxiety, were not comprehensively measured in the STEPS dataset, which may affect the completeness of cluster characterization. Future research should consider longitudinal studies to track the stability and transitions between clusters over time, which will enhance understanding of risk progression and inform timely interventions. Integration of more detailed behavioral, dietary, and psychosocial data could refine cluster profiles and identify additional modifiable factors.

The findings of this study have important implications for policy, practice, and research. The identification of distinct NCD risk clusters provides a valuable framework to prioritize and tailor public health policies and interventions. By targeting the specific risk profiles within each cluster, health authorities can design segmented prevention and management strategies that address the unique behavioral, metabolic, and socioeconomic factors influencing disease risk. For the SD cluster, interventions should prioritize smoking cessation, and behavioral counseling, particularly among older men and individuals with lower education. Early behavioral screening programs in schools and primary care center, combined with stronger tobacco and alcohol taxation policies, may also help reduce risk behaviors from a young age. For the DLP cluster, lipid screening, dietary counseling, weight-management programs, statin eligibility assessment may be most relevant. For the HTD cluster, integrated care models in urban clinics should prioritize concurrent management of hypertension, diabetes, and obesity including routine blood pressure and glucose monitoring. For the PA cluster, workplace wellness programs, occupational health screening, nutrition education, and smoking/alcohol prevention may be most relevant. Finally, for the HLR cluster, routine prevention and health-promotion strategies should aim to preserve favorable risk profiles and prevent transition to higher-risk clusters.

Future research can build on this clustering framework to deepen the understanding of NCD risk patterns and to develop personalized intervention approaches that improve health outcomes at the population level.

## Conclusion

In conclusion, our cluster analysis reveals five distinct co-occurrence pattern of behavioral and metabolic risk factors within the Iranian population. Rather than applying uniform prevention strategies to the whole population, these cluster-specific risk profiles allow health authorities to identify which risk factors should be prioritized and which demographic or socioeconomic groups are most susceptible to each pattern of risk. Although the HLR cluster was the largest single subgroup, the DLP and HTD clusters together accounted for nearly half of the study population, highlighting the importance of prioritizing metabolic risk prevention, screening, and management in related population within Iran’s NCD control programs. At the same time, behavioral risk factors should be addressed according to their distribution across clusters and vulnerable population groups. Continued surveillance and longitudinal research are needed to assess changes in cluster membership over time and to evaluate the effectiveness of cluster-specific prevention strategies.

## Supplementary Material

STROBE.docx

Supplementary Tables.docx

## Data Availability

The dataset used in this study is not publicly available, as it is the property of the National Institute for Health Research (NIHR) at Tehran University of Medical Sciences. Access to the data are restricted and subject to institutional policies. Requests for access should be directed to nihr@tums.ac.ir. Upon approval by NIHR, datasets utilized and/or examined in this study are available from the corresponding author upon reasonable request.
