# Supplementary material for: Clustering patterns of behavioral and metabolic risk factors for noncommunicable diseases in Iran: findings from a national STEPS survey
Source: Glob Health Action. 2026 Aug 10;19(1):2712185. doi: 10.1080/16549716.2026.2712185 (PMC13459308; doi:10.1080/16549716.2026.2712185)
Supplement: Supplementary file 1 [file ZGHA_A_2712185_SM8995.docx]

**Supplementary Table S1.** Comparing sociodemographic in individuals in include and exclude

|  | Include | Exclude |
| --- | --- | --- |
|  | n=16,618 | n=1,501 |
| Sex |  |  |
| Male | 9223 (55.5) | 1076 (71.7) |
| Female | 7395 (44.5) | 431 (28.7) |
| Age |  |  |
| 25 – 40 | 5650 (34.0) | 591 (39.4) |
| 41-60 | 7279 (43.8) | 663 (44.2) |
| Above 60 | 3689 (22.2) | 246 (16.4) |
| Residency |  |  |
| rural | 5351 (32.2) | 518 (34.5) |
| urban | 11267 (67.8) | 983 (65.5) |
| Education |  |  |
| zero | 2958 (17.8) | 246 (16.4) |
| 1-6 yr. | 4653 (28.0) | 440 (29.3) |
| 7-11 yr. | 3091 (18.6) | 291 (19.4) |
| 12 yr. and over | 5916 (35.6) | 524 (34.9) |
| Wealth index |  |  |
| poorest | 3540 (21.3) | 363 (24.2) |
| Class 1 | 3224 (19.4) | 290 (19.3) |
| Class 2 | 3589 (21.6) | 320 (21.3) |
| Class 3 | 3373 (20.3) | 290 (19.3) |
| Class 4 | 2892 (17.4) | 239 (15.9) |
| Marital status |  |  |
| single | 1280 (7.7) | 146 (9.7) |
| Married | 13810 (83.1) | 1270 (84.6) |
| Divorced/separate with partner | 382 (2.3) | 24 (1.6) |
| widow | 1147 (6.9) | 62 (4.1) |
| Occupation |  |  |
| employed | 6165 (37.1) | 411 (27.4) |
| Unpaid work | 7943 (47.8) | 950 (63.3) |
| Retired | 1662 (10.0) | 71 (4.7) |
| unemployed | 848 (5.1) | 69 (4.6) |
| Insurance |  |  |
| No | 1296 (7.8) | 119 (7.9) |
| Basic | 10719 (64.5) | 1060 (70.6) |
| complement | 4603 (27.7) | 323 (21.5) |
| General health | 71.4±20.6 | 70.8±21.6 |

**Supplementary Table S2.** Comparing characteristics in individuals in include and exclude

|  | Include | Exclude |
| --- | --- | --- |
|  | n=16,618 | n=1,501 |
| Physical activity | 7.6 ± 1.4 | 7.5 ± 1.4 |
| Nutrition score | -0.04 ± 0.99 | -0.04 ± 1.0 |
| Current smoking | 2.3 ± 9.8 | 1.6 ± 8.8 |
| Currently alcoholic | 0.19 ± 3.2 | 0.16 ± 2.39 |
| Salt | 9.7 ± 2.6 | 9.6 ± 2.9 |
| BMI | 27.3 ± 5.1 | 27.5 ± 5.4 |
| FBS | 104.5 ± 34.8 | 103.8 ± 35.9 |
| SBP | 127.6 ± 19.0 | 126.6 ± 19.3 |
| DBP | 79.0 ± 11.4 | 78.9 ± 11.2 |
| TC | 171.9 ± 37.0 | 172.0 ± 37.6 |
| HDL | 42.2 ± 10.3 | 43.4 ± 10.5 |
| LDL | 99.8 ± 31.9 | 99.5 ± 32.0 |

BMI, Body Mass Index; FPG, Fasting Plasma Glucose; SBP, Systolic Blood Pressure; DBP, Diastolic Blood Pressure; TC, Total Cholesterol; HDL, High-Density Lipoprotein; LDL, Low-Density Lipoprotein; TG, Triglycerides.

**Supplementary Table S3.** The scoring of variables used in constructing the nutrition score

| Variable | Categories | Score |
| --- | --- | --- |
| Mean daily fruit servings* | ≥4 servings | 1 |
|  | 3 ≤ and < 4 servings | 0.75 |
|  | 2 ≤ and < 3 servings | 0.5 |
|  | 1 ≤ and < 2 serving | 0.25 |
|  | <1 servings | 0 |
| Mean daily vegetables servings* | ≥4 servings | 1 |
|  | 3 ≤ and < 4 servings | 0.75 |
|  | 2 ≤ and < 3 servings | 0.5 |
|  | 1 ≤ and < 2 serving | 0.25 |
|  | <1 servings | 0 |
| Frequency of consuming fish | Less than 1 time/month or never | 0 |
|  | 1-3 times/month | 0.2 |
|  | 1-3 times/week | 0.4 |
|  | 4-6 times/week | 0.6 |
|  | 1 or 2 times a day | 0.8 |
|  | More than 3 times a day | 1 |
| Frequency of consuming whole grains (including brown rice, grain bread, barley bread, and pasta) | Less than 1 time/month or never | 0 |
|  | 1-3 times/month | 0.2 |
|  | 1-3 times/week | 0.4 |
|  | 4-6 times/week | 0.6 |
|  | 1 or 2 times a day | 0.8 |
|  | More than 3 times a day | 1 |
| Frequency of consuming red meat | Less than 1 time/month or never | 0 |
|  | 1-3 times/month | 0.2 |
|  | 1-3 times/week | 1 |
|  | 4-6 times/week | 0.8 |
|  | 1 or 2 times a day | 0.6 |
|  | More than 3 times a day | 0.4 |
| Frequency of sugar-sweetened beverages consumption | Less than 1 time/month or never | 1 |
|  | 1-3 times/month | 0.8 |
|  | 1-3 times/week | 0.6 |
|  | 4-6 times/week | 0.4 |
|  | 1 or 2 times a day | 0.2 |
|  | More than 3 times a day | 0 |
| Frequency of eating processed meats | Less than 1 time a month or never | 1 |
|  | 1 to 3 times a month | 0.8 |
|  | 1 to 3 times a week | 0.6 |
|  | 4 to 6 times a week | 0.4 |
|  | 1 or 2 times a day | 0.2 |
|  | More than 3 times a day | 0 |
| number of meals per day | 1 time per day | 0 |
|  | 2 times per day | 0.5 |
|  | 3 times per day or more | 1 |
| number of snacks per day | 0 time per day | 0 |
|  | 1 time per day | 0.33 |
|  | 2 times per day | 0.66 |
|  | 3 times per day or more | 1 |
| days of eating breakfast per week | Always | 1 |
|  | 5 to 6 days | 0.75 |
|  | 3 to 4 days | 0.5 |
|  | 1 to 2 days | 0.25 |
|  | Never | 0 |
| consumption of dairy products (including milk, yogurt, and cheese) | Less than 1 time a month or never | 0 |
|  | 1 to 3 times a month | 0.2 |
|  | 1 to 3 times a week | 0.4 |
|  | 4 to 6 times a week | 0.6 |
|  | 1 or 2 times a day | 0.8 |
|  | More than 3 times a day | 1 |
| type of dairy | Low fat | 1 |
|  | High fat | 0.66 |
|  | 2.5% fat | 0.33 |
|  | Native dairy | 0 |
| nuts and seeds consumption | Less than 1 time a month or never | 0 |
|  | 1 to 3 times a month | 0.2 |
|  | 1 to 3 times a week | 0.4 |
|  | 4 to 6 times a week | 0.6 |
|  | 1 or 2 times a day | 0.8 |
|  | More than 3 times a day | 1 |

Mean daily fruit and vegetables serving were calculated by multiplying the reported number of servings consumed per day by the number of days they were consumed in a week, and then dividing the result by 7. This provides an average daily intake over the course of a week.

**Supplementary Table S4.** Mean values of different subtypes of physical activity across the Five Identified Clusters.

|  | **cluster 1 (SD)**  **n=509** | **cluster 2 (HLR)**  **n=6,700** | **cluster 3 (PA)**  **n=1,101** | **cluster 4 (DLP)**  **n=4,326** | **cluster 5 (HTD)**  **n=3,982** |
| --- | --- | --- | --- | --- | --- |
| Vigorous work | 1019.4  [655.9,1382.9] | 739.9  [655.2,824.8] | 7953.8  [7132.1 ,8775.7] | 603.9  [513.2 ,694.7] | 608.2  [507.6 ,708.7] |
| moderate work | 1038.4  [768.94 ,1308.0] | 776.7  [703.04 ,850.51] | 3337.9  [2937.24 ,3738.67] | 789.4  [701.61 ,877.23] | 723.5  [639.58 ,807.57] |
| Transport | 689.3  [560.52 ,818.22] | 637.9  [594.50 ,681.41] | 1737.9  [1493.01 ,1982.83] | 676.6  [626.18 ,727.11] | 623.8  [578.08 ,669.52] |
| vigorous recreational activities | 136.0  [64.72 ,207.44] | 223.4  [194.43 ,252.51] | 1019.5  [781.38 ,1257.76] | 193.0  [160.18 ,225.99] | 169.3  [137.21 ,201.36] |
| moderate recreational activities | 102.5  [14.65 ,190.49] | 94.8  [83.18 ,106.46] | 338.5  [146.40 ,530.66] | 95.3  [75.90 ,114.67] | 76.4  [58.29 ,94.60] |

SD indicates Smokers Drinkers; HLR, Healthy and Low Risk; PA, Physically Active; DLP, Dyslipidemic; HTD, Hypertensive Diabetics.

**Supplementary Table S5.** Mean log-transformed MET-min/week values for total physical activity and physical activity subtypes across the five identified clusters.

| **Physical activity domain** | **Cluster 1 (SD)** | **Cluster 2 (HLR)** | **Cluster 3 (PA)** | **Cluster 4 (DLP)** | **Cluster 5 (HTD)** |
| --- | --- | --- | --- | --- | --- |
| Total physical activity | 7.78 [7.62, 7.95] | 7.43 [7.39, 7.48] | 9.08 [8.99, 9.18] | 7.44 [7.39, 7.50] | 7.40 [7.34, 7.46] |
| Vigorous work | 8.23 [7.95, 8.51] | 7.99 [7.91, 8.08] | 9.31 [9.23, 9.40] | 7.80 [7.68, 7.92] | 7.96 [7.83, 8.09] |
| Moderate work | 7.73 [7.51, 7.94] | 7.34 [7.28, 7.41] | 8.28 [8.17, 8.38] | 7.41 [7.33, 7.49] | 7.41 [7.32, 7.50] |
| Transport-related activity | 6.83 [6.67, 6.98] | 6.60 [6.56, 6.64] | 7.25 [7.14, 7.36] | 6.67 [6.62, 6.72] | 6.68 [6.63, 6.73] |
| Vigorous recreational activity | 7.52 [7.06, 7.98] | 7.27 [7.20, 7.35] | 7.79 [7.63, 7.96] | 7.24 [7.14, 7.34] | 7.17 [7.06, 7.28] |
| Moderate recreational activity | 6.59 [6.24, 6.95] | 6.41 [6.33, 6.50] | 6.93 [6.75, 7.12] | 6.48 [6.36, 6.60] | 6.46 [6.33, 6.59] |

Values are presented as mean log-transformed MET-min/week [95% confidence interval]. SD indicates Smokers Drinkers; HLR, Healthy and Low Risk; PA, Physically Active; DLP, Dyslipidemic; HTD, Hypertensive Diabetics; MET, metabolic equivalent of task.

**Supplementary Table S6.** Univariable Multinomial Logistic Regression Analysis of Predictors for NCD Risk Cluster Membership.

| variables | **cluster 1 (SD)** | **cluster 2 (HLR)** | **cluster 3 (PA)** | **cluster 4 (DLP)** | **cluster 5 (HTD)** |
| --- | --- | --- | --- | --- | --- |
| Sex  (female) |  |  |  |  |  |
| male | 1.63 (1.33,2.00) | **1 (Reference)** | 1.87 (1.62,2.16) | 0.94 (0.87,1.03) | 1.05 (0.96,1.15) |
| Age (25-40) |  |  |  |  |  |
| 41-60 | 3.96 (2.99,5.24) | 1 (Reference) | 1.20 (1.03,1.40) | 2.40 (2.17,2.65) | 2.70 (2.43,3.01) |
| above 60 | 4.98 (3.66,6.78) | 1 (Reference) | 0.74 (0.59,0.92) | 2.50 (2.22,2.82) | 3.44 (3.04,3.90) |
| Residency  ( rural |  |  |  |  |  |
| urban | 0.72 (0.58,0.89) | 1 (Reference) | 0.46 (0.40,0.53) | 0.95 (0.86,1.04) | 1.08 (0.98,1.19) |
| Education ( Zero) |  |  |  |  |  |
| 1-6 yr. | 0.77 (0.59,1.00) | 1 (Reference) | 1.13 (0.90,1.41) | 0.84 (0.74,0.97) | 0.74 (0.65,0.85) |
| 7-11 yr. | 0.40 (0.29,0.55) | 1 (Reference) | 1.15 (0.91,1.45) | 0.62 (0.54,0.72) | 0.54 (0.46,0.62) |
| 12 yr. and over | 0.19 (0.14,0.26) | 1 (Reference) | 0.57 (0.45,0.71) | 0.58 (0.51,0.65) | 0.41 (0.36,0.47) |
| Wealth index ( Poorest) |  |  |  |  |  |
| Class 1 | 0.60 (0.45,0.81) | 1 (Reference) | 0.79 (0.64,0.97) | 0.94 (0.82,1.08) | 0.87 (0.76,1.00) |
| Class 2 | 0.71 (0.53,0.94) | 1 (Reference) | 0.89 (0.73,1.10) | 1.00 (0.87,1.14) | 1.00 (0.87,1.14) |
| Class 3 | 0.54 (0.40,0.72) | 1 (Reference) | 0.69 (0.56,0.86) | 1.03 (0.90,1.17) | 0.93 (0.81,1.07) |
| Class 4 | 0.30 (0.21,0.44) | 1 (Reference) | 0.48 (0.37,0.61) | 0.99 (0.87,1.14) | 0.79 (0.69,0.91) |
| Marital Status  (Single) |  |  |  |  |  |
| Married | 1.58 (1.04,2.40) | 1 (Reference) | 1.98 (1.45,2.72) | 1.15 (0.98,1.34) | 1.44 (1.22,1.71) |
| Divorced/separate with partner | 1.30 (0.61,2.77) | 1 (Reference) | 0.97 (0.51,1.84) | 1.09 (0.80,1.49) | 1.14 (0.82,1.58) |
| Widow | 1.74 (0.99,3.08) | 1 (Reference) | 0.64 (0.37,1.10) | 1.84 (1.48,2.28) | 1.96 (1.56,2.48) |
| Occupation (employee ) |  |  |  |  |  |
| Unpaid work | 0.92 (0.73,1.15) | 1 (Reference) | 0.59 (0.51,0.69) | 1.30 (1.19,1.43) | 1.30 (1.18,1.43) |
| Retired | 1.51 (1.07,2.13) | 1 (Reference) | 0.38 (0.27,0.53) | 1.60 (1.37,1.86) | 1.98 (1.70,2.31) |
| Unemployed | 1.30 (0.85,2.00) | 1 (Reference) | 0.47 (0.32,0.68) | 1.20 (0.98,1.48) | 1.23 (1.00,1.51) |
| Insurance  (no) |  |  |  |  |  |
| Basic | 0.96 (0.68,1.36) | 1 (Reference) | 1.15 (0.90,1.47) | 1.12 (0.96,1.30) | 1.10 (0.94,1.29) |
| Complementa | 0.80 (0.54,1.18) | 1 (Reference) | 0.50 (0.37,0.67) | 1.30 (1.10,1.53) | 1.43 (1.20,1.69) |

SD indicates Smokers Drinkers; HLR, Healthy and Low Risk; PA, Physically Active; DLP, Dyslipidemic; HTD, Hypertensive Diabetics.

**Supplementary Table S7.** p-values for the multivariable multinomial logistic regression analysis of predictors of NCD risk cluster membership

| **Variable** | **Category** | **SD vs HLR** | **PA vs HLR** | **DLP vs HLR** | **HTD vs HLR** |
| --- | --- | --- | --- | --- | --- |
| Sex | Male | <0.001 | <0.001 | 0.067 | 0.012 |
| Age | 41–60 | <0.001 | 0.243 | <0.001 | <0.001 |
| Age | Above 60 | <0.001 | 0.052 | <0.001 | <0.001 |
| Residency | Urban | 0.801 | <0.001 | 0.163 | 0.005 |
| Education | 1–6 yr. | 0.262 | 0.392 | 0.787 | 0.008 |
| Education | 7–11 yr. | <0.001 | 0.333 | 0.141 | <0.001 |
| Education | ≥12 yr. | <0.001 | <0.001 | 0.113 | <0.001 |
| Wealth index | Class 1 | 0.024 | 0.458 | 1.000 | 0.266 |
| Wealth index | Class 2 | 0.210 | 0.392 | 0.106 | 0.144 |
| Wealth index | Class 3 | 0.085 | 0.182 | 0.014 | 0.387 |
| Wealth index | Class 4 | 0.002 | 0.025 | 0.033 | 0.390 |
| Marital status | Married | 0.016 | 0.002 | <0.001 | 0.002 |
| Marital status | Divorced/separated | 0.185 | 1.000 | 0.056 | 0.029 |
| Marital status | Widow | 0.004 | 0.251 | 0.116 | 0.001 |
| Occupation | Unpaid work | 0.165 | 0.903 | <0.001 | 0.005 |
| Occupation | Retired | 0.536 | 0.009 | 0.140 | 0.017 |
| Occupation | Unemployed | 0.286 | <0.001 | 0.485 | 0.786 |
| Insurance | Basic | 0.327 | 0.818 | 0.908 | 0.908 |
| Insurance | Basic + Complementary | 0.148 | 0.003 | 1.000 | 0.325 |

Cluster 2, Healthy and Low Risk (HLR), was used as the reference cluster. Reference categories for predictors were female for sex, 25–40 years for age, rural for residency, zero years for education, poorest for wealth index, single for marital status, employed for occupation, and no insurance for insurance status. SD indicates Smokers Drinkers; PA, Physically Active; DLP, Dyslipidemic; HTD, Hypertensive Diabetics.
